# VEXAS syndrome: complete molecular remission after hypomethylating therapy

**DOI:** 10.1007/s00277-023-05611-w

**Published:** 2024-01-12

**Authors:** Katja Sockel, Katharina Götze, Christina Ganster, Marius Bill, Julia-Annabell Georgi, Ekaterina Balaian, Martin Aringer, Karolin Trautmann-Grill, Maria Uhlig, Martin Bornhäuser, Detlef Haase, Christian Thiede

**Affiliations:** 1 Medical Clinic and Policlinic I, University Hospital Dresden, TU Dresden, Dresden, Germany; 2 German Cancer Consortium (DKTK), CHOICE Consortium, Partner Sites, MunichDresden, Germany; 3 Department of Medicine III, Klinikum rechts der Isar, Technical University of Munich (TUM), Munich, Germany; 4 German Cancer Research Center (DKFZ), Heidelberg, Germany; 5 Clinics of Hematology and Medical Oncology, University Medical Center Göttingen, Göttingen, Germany; 6 Division of Rheumatology, Department of Medicine III, University Medical Center and Faculty of Medicine Carl Gustav Carus at the TU Dresden, Dresden, Germany; 7 National Center for Tumor Diseases (NCT/UCC), Dresden, Germany; 8 AgenDix GmbH, Dresden, Germany

**Keywords:** VEXAS, Hypomethylating therapy, Azacitidine, Molecular remission

## Abstract

The VEXAS syndrome, a genetically defined autoimmune disease, associated with various hematological neoplasms has been attracting growing attention since its initial description in 2020. While various therapeutic strategies have been explored in case studies, the optimal treatment strategy is still under investigation and allogeneic cell transplantation is considered the only curative treatment. Here, we describe 2 patients who achieved complete molecular remission of the underlying *UBA1* mutant clone outside the context of allogeneic HCT. Both patients received treatment with the hypomethylating agent azacitidine, and deep molecular remission triggered treatment de-escalation and even cessation with sustained molecular remission in one of them. Prospective studies are necessary to clarify which VEXAS patients will benefit most from hypomethylating therapy and to understand the variability in the response to different treatment strategies.

## Introduction

Since its first description in 2020, the VEXAS syndrome (vacuoles, E1 enzyme, X-linked, autoinflammatory, somatic), a genetically defined auto-inflammatory disease associated with hematological abnormalities, has gained increasing attention [1]. With 1:4000–1:14000 cases in the general population [2], the disease prevalence is much higher than initially expected. Clinical inflammatory manifestations are variable and often resistant to conventional immunosuppressive treatments. The majority of patients exhibit hematological abnormalities, including myelodysplastic neoplasm (MDS), macrocytic anemia, monoclonal gammopathy, and multiple myeloma [3]. Disease pathogenesis is linked to a loss of UBA1 function by acquired somatic mutations, most frequently affecting methionine 41 (p.M41) of the *UBA1* gene, which codes for the E1 enzyme that regulates protein ubiquitination. Reduced ubiquitination mediates activation of the innate immune system and synthesis of proinflammatory cytokines, such as interleukin (IL)-1, IL-6, IL-8, interferon, and tumor necrosis factor alpha [1], which contribute to the inflammatory phenotype of VEXAS syndrome.

Various therapeutic strategies have been described in case studies, ranging from steroids over azacitidine [4, 5] to ruxolitinib [6] and allogeneic hematopoietic cell transplantation (alloHCT) [7, 8]. The latter has been considered the only curative treatment option so far. Here we report for the first time complete molecular clearance of the underlying *UBA1* mutation in two VEXAS patients (Table 1) who underwent treatment with the hypomethylating agent azacitidine (5-Aza), leading to treatment de-escalation. Written informed consent for clinical follow-up and detailed molecular diagnostics were obtained from both patients before treatment start.

**Table 1:** Patient characteristics at baseline

Variable	Patient 1	Patient 2
Clinical data		
Age	72 y	68 y
Symptoms	Intermittent fever, arthralgia, nodular erythema, lymph node enlargement, macrocytic anemia, night sweats, and 10 kg of weight loss	Intermittent fever, progressive arthralgias, weight loss, Sweet syndrome, thrombosis, macrocytic anemia
Blood counts		
Hemoglobin	8.7 g/dl	9.7 g/dl
MCV	97 fl	97 fl
Platelets	304 g/l	120 g/l
Neutrophils	0.76 g/l	0.69 g/l
*UBA1* mutation (VAF)		
PB	83.15%	59.6%
BM	62%	29.43%
Bone marrow		
Cytomorphology	Dysplasia, 6% blasts	Dysplasia, 7% blasts
Genetics	del (20q)	46;XY
Molecular genetics (other than *UBA1*)	No additional mutations	Low-level (VAF 0.9%) *TET2 mutation* (p.Ser1708fs)
IPSS-M	Moderate-high	Very low
Prior treatment	Prednisolone 1 mg/kg, colchicine, dapsone, and interleukin-1 inhibitor	Prednisolone 1 mg/kg, MTX, azathioprine, and interleukin-1 inhibitor

## Case description

### Patient 1

*A 72-year-old man presented with a 6-month history of recurrent fever, arthralgias, nodular erythema, enlarged mesenterial lymph nodes, macrocytic anemia, night sweats, and 10 kg of weight loss. Lymph node biopsy showed no pathological findings. He was started on prednisolone 1 mg/kg and initially responded well but remained steroid-dependent at 20 mg/day. Colchicine, dapsone, and interleukin-1 blockade had no longer-lasting benefit. Instead, the patient developed further deterioration with pneumonitis and respiratory impairment, leading to wheelchair mobility.*

*A bone marrow biopsy in 3/2019 revealed MDS with increased blasts (MDS-IB1) and del(20q), while molecular alterations were not detected (note that UBA1 was not analyzed at this time). According to the Molecular International Prognostic Scoring System (IPSS-M), a moderate-high-risk MDS was diagnosed, with autoinflammatory features. Due to severe symptoms and steroid dependence, treatment with the hypomethylating agent (HMA) azacitidine 75 mg subcutaneously on days 1–7 every 28 days was initiated. The autoinflammatory symptoms resolved after 2 cycles, and blood counts normalized after 4 cycles. Notably, steroids could be discontinued during the first 2 cycles.*

*After the description of VEXAS syndrome in 2020 *[1]*, we retrospectively sequenced DNA from initial diagnosis by next-generation sequencing (NGS) and identified an UBA1 (c.118-1G* > *C) mutation. Longitudinal molecular follow-up using ultradeep NGS with a detection limit of 0.1% *[9]* revealed complete clearance of this UBA1-mutant clone in the bone marrow 6 months after treatment start. At this timepoint, also MDS del(20q) was in complete cytogenetic remission.*

**Fig. 1:**
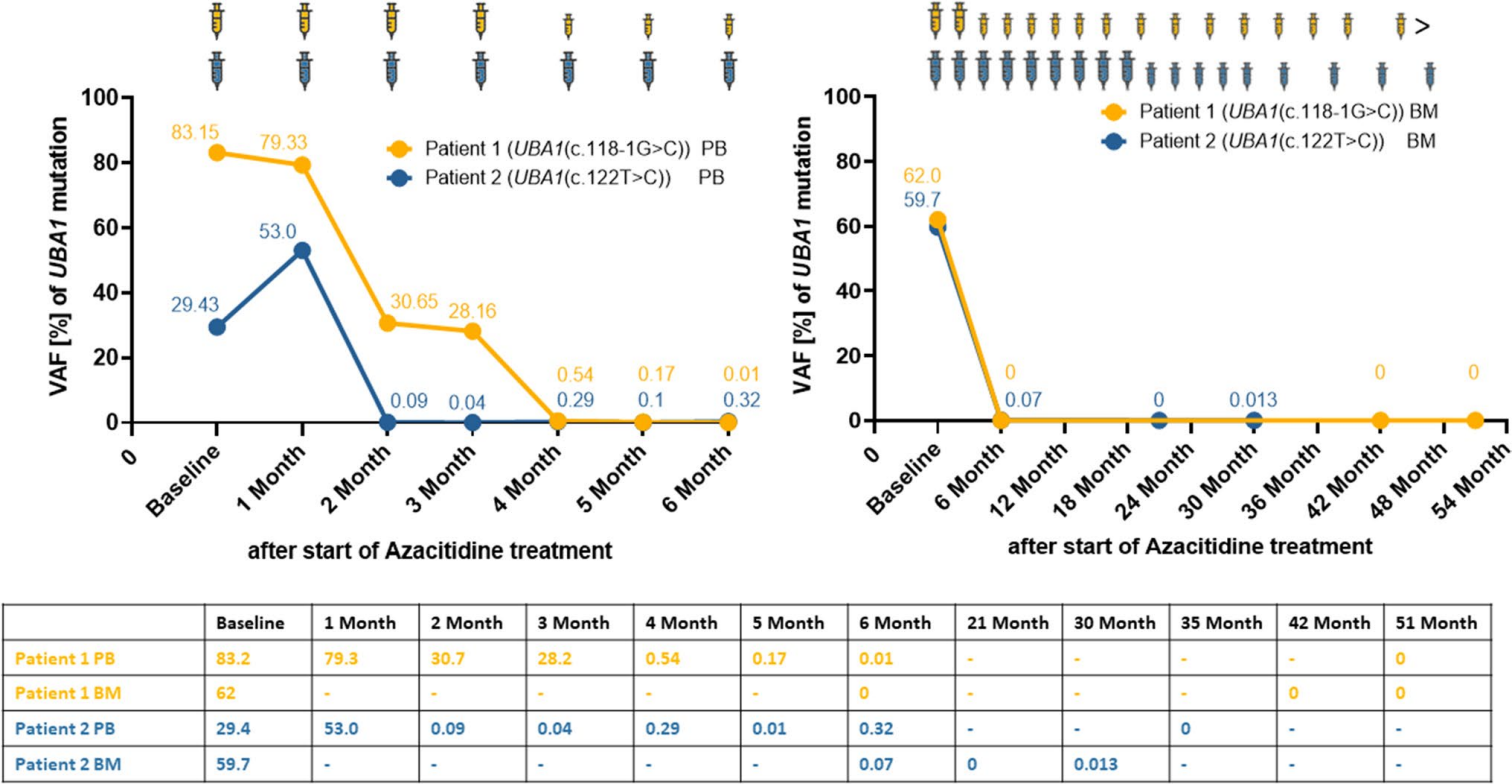
Variant allele frequencies (VAF) of the *UBA1* mutation during azacitidine treatment. Left: *UBA1* VAF in CD34-selected peripheral blood (pB) during the first 6 treatment cycles. Right: *UBA1* VAF in unsorted BM cells. Ultradeep NGS with a detection limit of 0.1%^9^ was used for longitudinal molecular follow-up. Patient 1 (yellow) achieved complete eradication of the *UBA1* clone 6 months after azacitidine start.  Follow-up assessments in patient 2 (blue) identified complete molecular remission at month 21 after treatment start

*Due to the rapid response, 5-Aza treatment was reduced to 5 days and cycle administration extended to every 6–8 weeks. Treatment was ultimately discontinued in March 2023. As of October 2023, the patient continues to demonstrate ongoing hematological, clinical, and molecular remission (see *Fig. 1*).*

### Patient 2

*In 2019, a 68-year-old male patient was referred with progressive arthralgias, weight loss, and recurrent fever episodes over the past 2 years. He had a history of spontaneous deep vein thrombosis of the leg 2 years before (low molecular weight heparins were already stopped) and mild macrocytic anemia. High-dose steroids provided temporary relief, while the use of methotrexate, azathioprine, and interleukin-1 blockade, which were initially prescribed for suspected polymyalgia rheumatica, did not yield significant improvement. At the time of presentation, high fever episodes were present, along with erythematous subcutaneous nodules, confirmed as Sweet syndrome through biopsy. A PET-CT scan revealed no signs of malignancy or vasculitis but showed significant bone marrow activation. Bone marrow biopsy revealed MDS-IB1 (WHO 2022), without cytogenetic abnormalities. Molecular analysis identified a low level TET2 mutation (VAF 0.9%). Diagnosis of MDS with very low risk (according to IPSS-M) and autoinflammatory symptoms was made and azacitidine 75mg on days 1-7 subcutaneously was initiated due to severe clinical course. HMA treatment led to a rapid clinical response with complete regression of the rash and joint symptoms. Steroids could be tapered off during the first 2 cycles.*

*NGS resequencing detected the UBA1 p.M41T (c.122 T* > *C) mutation confirming VEXAS syndrome and follow-up assessments identified complete molecular remission at month 21 after treatment start, in accordance with complete molecular remission of the underlying MDS. This observation prompted dose reduction of 5-Aza to 5-day cycles every 8 weeks. However, at the 30-month follow-up, a low-level UBA1 mutation (VAF 0.013%) temporarily reappeared. Since then, the patient has consistently been in complete clinical, hematological, and molecular remission.*

## Results and discussion

Longitudinal molecular monitoring of the *UBA1-*mutated clone during 5-Aza therapy revealed complete molecular remission 6 months (patient 1) and 21 months (patient 2) after treatment start.

To our knowledge, this is the first report describing complete molecular clearance of the *UBA1*-mutant clone in two VEXAS patients outside the context of alloHCT. While some authors have reported the suppression of the *UBA1*-mutant clone after HMA therapy [10], complete molecular clearance had not been observed until yet.

To further corroborate the depth of remission, we performed molecular monitoring using ultradeep NGS with a detection limit of 0.1% (median coverage > 100,000 reads) in CD34-selected peripheral blood (pB) (Fig. 1), which has a calculated overall sensitivity of 0.01–0.001% [9], indicating a very deep molecular clearance of the mutation.

Azacitidine, a hypomethylating agent approved for high-risk MDS and commonly recommended for patients with MDS and autoinflammatory features [4], has already been successfully used to treat autoinflammatory symptoms in VEXAS syndrome [4, 5]. Besides direct cytotoxic effects, azacitidine modulates the cytokine profile with downregulation of proinflammatory cytokines and impacts the bone marrow microenvironment [11], which might contribute to its positive effect on autoinflammatory sequelae.

In our patients, azacitidine treatment not only resulted in clinical and hematological remissions but also induced complete molecular eradication of the underlying *UBA1*-mutated clone, below the detection limit of our method, clearly documenting the profound and specific effect on the mutant population.

Notably, steroids could be tapered within 2 months of treatment indicating that 5-Aza alone was sufficient to maintain remission in these patients. Nevertheless, the underlying mechanism by which 5-Aza impacts the *UBA1* clonal burden is not clear, although direct cytotoxic effects have been described and a synthetic lethal interaction with the *UBA1* clone has been speculated [12]. Prospective studies are necessary to clarify which VEXAS patients will benefit mostly from hypomethylating therapy, since different mutations may lead to differences in prognosis, phenotype, or even treatment responses.

To date, the three most common mutations in VEXAS syndrome impact methionine 41 in exon 3 of the *UBA1 gene*, namely p.M41Thr (c.122 T > C), p.M41Val (c.121A > G), and p.M41Leu (c.121A > C) [1]. In addition, non-M41 gene mutations have been described, such as splice site mutations at exon 3 (c.118-2A > C, c.118-1G > C, c.118-9_118-2del) and mutations affecting codon 56 (c.167C > T) [13, 14]. In our case, patient 2 exhibited the common p.M41Thr mutation, while patient 1 who successfully discontinued HMA therapy had the *UBA1* (c.118-1G > C) mutation, affecting the splice region at exon 3. Recently, Georgin-Lavialle [15] has described that the *UBA1* p.M41Leu mutation is associated with a milder phenotype and a better 5-year survival rate compared to p.M41Val and p.M41Thr. However, a clear genotype-phenotype correlation, or even a correlation of certain mutations with responses to specific treatment strategies, has not yet been established.

The deep molecular response observed in our patients prompted us to consider treatment de-escalation with dose reduction of 5-Aza from 7 to 5 days, followed by treatment cycle extension to every 6 to 8 weeks, and ultimately treatment discontinuation in patient 1 after 44 months. Notably, he remained in sustained complete molecular remission 6 months after treatment discontinuation. However, the second patient temporarily exhibited a very low-level *UBA1* mutation at month 30 during 5-Aza therapy, suggesting the persistence of *UBA1*-mutated cells in the quiescent stem cell pool, similar to the “leukemia stem cell persistence” observed in chronic myeloid leukemia [16]. Further prospective studies are necessary to clarify whether low-level minimal residual disease (MRD) or even complete clearance can guide safe treatment de-escalation. Close MRD monitoring in those patients is essential. Indeed, NGS in CD34+ -enriched pB cells provides a more sensitive detection of MRD compared to unsorted BM cells, as recently described [9, 17].

Co-mutations in VEXAS syndrome predominantly involve epigenetic regulators and splicing factors. DNMT3A and TET2, well-known for their association with inflammatory conditions, were the most commonly observed [18]. We also detected a low-level *TET2* co-mutation in one of our patients, which may be indicative of clonal hematopoiesis due to its low VAF. However, due to multilineage dysplasia, increased blast cell count, and cytopenia, the diagnosis of MDS was established.

Of note, in both patients, 5-Aza not only addressed the VEXAS syndrome but also achieved complete remission of the associated MDS disease, including hematological, cytogenetic (case 1), and molecular (case 2) aspects. Neither additional myeloid mutations nor novel cytogenetic alterations could be observed during the follow-up phase. The exact relationship between the UBA1-mutant clone and the initiation of MDS is yet to be determined. It remains uncertain whether the myeloid neoplasm is primarily driven by the UBA1-mutant clone or whether the highly inflammatory microenvironment promotes clonal selection.  

In addition to azacitidine, further treatment approaches have been described in small VEXAS cohorts. Among them, the JAK1/2 inhibitor ruxolitinib is considered one of the most promising, which has successfully alleviated symptoms of VEXAS syndrome; however, no molecular remissions have been reported thus far. Indeed, a recent publication reported an increase in UBA1 burden under ruxolitinib, despite a highly favorable clinical response [8].

In conclusion, detailed molecular monitoring of two VEXAS syndrome patients who underwent HMA therapy revealed complete eradication of the *UBA1* clone along with significant clinical and hematologic responses. Thus, hypomethylating agents might be an interesting alternative, at least for a proportion of patients, not eligible for alloHCT. Clearly, this report is based on a limited number of cases, and prospective studies are required to validate these findings and identify which VEXAS patients will benefit most from HMA.

## Data Availability

No datasets were generated or analysed during the current study.
